# Dielectric and Interface Properties of Aluminum-Laminated Lanthanum Oxide on Silicon for Nanoscale Device Applications

**DOI:** 10.3390/nano15130963

**Published:** 2025-06-21

**Authors:** Hei Wong, Weidong Li, Jieqiong Zhang, Jun Liu

**Affiliations:** 1 Department of Electrical Engineering, City University of Hong Kong, Hong Kong, China; 2 Yangtze Memory Technologies Co., Ltd., East Lake High-Tech Development Zone, Wuhan 430078, China; 3 Hubei Jiu Feng Shan Laboratory, Wuhan 430074, China

**Keywords:** rare-earth oxide, gate dielectric, interface, XPS, lanthanum aluminate

## Abstract

By embedding an aluminum-laminated layer within La_2_O_3_ thin films and subjecting them to high-temperature rapid thermal annealing, a La_2_O_3_/LaAl*_x_*O*_y_*/La_2_O_3_ sandwich dielectric was formed. This structure enhances the interface properties with both the silicon substrate and the metal gate electrode, improving current conduction. Comprehensive analysis using X-ray Photoelectron Spectroscopy (XPS) revealed that this novel process not only facilitates the formation of a high-quality lanthanum aluminate layer, as indicated with Al 2p peak at 74.5 eV, but also effectively suppresses silicate layer growth, as supported by the weak Si-O signal from both the Si 2s (153.9 eV) and O 1s (533 eV) peaks at the dielectric/Si interface in the Al-laminated samples. Fourier Transform Infrared (FTIR) spectroscopy revealed a significant reduction in the OH absorption peak at 3608 cm^−1^ OH-related band centered at 3433 cm^−1^. These improvements are attributed to the aluminum-laminated layer, which blocks oxygen and hydroxyl diffusion, the LaAl*_x_*O*_y_* layer scavenging interface silicon oxide, and the consumption of oxygen during LaAl*_x_*O*_y_* formation under thermal annealing. Electrical measurements confirmed that the dielectric films exhibited significantly lower interface and oxide trap densities compared to native La_2_O_3_ samples. This approach provides a promising method for fabricating high-quality lanthanum-based gate dielectric films with controlled dielectric/substrate interactions, making it suitable for nano-CMOS and memristive device applications.

## 1. Introduction

To maintain proper control of the channel current of the nanoscale complementary metal–oxide-semiconductor (CMOS) transistor with a down-scaled gate biasing voltage, a subnanometer equivalent oxide thickness (EOT) gate dielectric is indispensable [1,2,3,4]. Hafnium-based high-k gate dielectrics have been employed since the 45 nm technology node and the EOT of the gate dielectric film was scaled down to the 1 nm range [5,6,7]. However, gate dielectric technology has lagged far behind the development of CMOS technology in the last decade. The gate oxide EOT of the 5 nm technology or the 10 nm gate length device is still around 0.7 to 0.8 nm, which is much thicker than the minimum thickness suggested by Dennard’s constant-field scaling rule [8]. The main challenges are the thermal instability and interface of the high-k metal oxide on the silicon substrate. Moreover, the hafnium-based gate dielectric can only provide a k value of about 15 when considering the low-k interfacial layer, which is not suitable for half-nanometer EOT gate dielectric applications. A comprehensive search for higher-dielectric-constant rare-earth (RE) metal oxides has been conducted [9,10,11,12,13,14,15,16,17,18,19,20,21]. Among the many rare-earth metal oxides, lanthanum oxide, a dielectric material with a value of about 27 and band offsets with silicon over 2 eV, is one of the candidates [9,10]. However, most of the problems encountered in hafnium oxide, such as thermal instability and a low-k transition layer on the interface, remain key challenges, in addition to the hygroscopic nature of the lanthanum oxide [2,3]. The hygroscopic issue was partially solved with in situ high-k/metal gate deposition [3,10,11,13,14]. The material instability issues were also mitigated with metal doping, which includes rare-earth metals such as Y, Gd, or Sc, which can alter the crystal structure to mitigate the hygroscopicity and interfacial instability [4,14], or non-RE metals, such as Al, Zr, or Hf, which can reduce oxygen vacancies to a certain extent [2]. This may still be an issue regarding the long-term stability of the devices [2,3].

Lanthanum alumina is widely recognized as a highly stable dielectric [17]. Crystalline LaAlO_3_ has long been valued for its thermal stability, making it a suitable material for optical coatings. The LaAlO_3_/SrTiO_3_ structure has been extensively studied for its superconducting properties, which arise from the formation of a two-dimensional electron gas at the mismatched interface [18,19]. The direct deposition of LaAlO_3_ onto silicon has been shown to yield a sharp interface, improved thermal stability, and a reduced leakage current [22]. Additionally, LaAlO_3_ films have been employed as electrodes in methane-fueled solid oxide fuel cells [23] and have promising applications in memristors, a technology poised to significantly impact future memory systems and neuromorphic computing [24,25]. Notably, an oxidative Al_2_O_3_ layer was found to enhance the resistive switching properties of AlN-based memristors [25,26]. However, obtaining a high interface state density remains a challenge due to mismatch-induced defects. After vacuum annealing at 600 °C, substantial interface growth was observed, attributed to oxidation from residual oxygen and hydroxyl groups. This finding aligns with our hypothesis that oxygen hydroxy diffusion plays a role in enhancing the interface of the La_2_O_3_/Si structure. The resulting interfacial oxide layer facilitates calcination reactions between La_2_O_3_ and SiO_2_, leading to the formation of lanthanum silicates. Furthermore, the ternary LaAlO_3_ system, along with the substrate silicon and interfacial silicon oxide, may be more susceptible to interface reactions at elevated temperatures, as dictated by thermodynamic and bonding chemistry considerations.

In this work, we aim to develop a novel LaAlO_3_ formation process that effectively suppresses interface layer growth. By embedding an aluminum lamination layer within the La_2_O_3_ film, we successfully mitigated interface growth during thermal annealing, resulting in a lower interface trap density, reduced flatband shift, and a minimized leakage current. X-ray photoelectron spectroscopy (XPS) analysis reveals that, after annealing at 700 °C in N_2_ for 30 min, the Al layer transforms into lanthanum aluminate, significantly reducing the bulk oxygen content. While interface lanthanum silicate is still present, its thickness remains below 1 nm, which is sufficient to avoid significant interface trap density while preserving the equivalent oxide thickness (EOT).

This discovery not only advances the development of high-quality high-k dielectric technology but also has implications for interface property control in LaAlO_3_/SrTiO_3_ structures. The details of our experimental procedures are presented in the next section. Section 3 provides a comprehensive analysis of the film and interface structures of both as-deposited and thermally annealed samples, utilizing XPS measurements. Additionally, we examine the electrical properties of MOS capacitors through capacitance-voltage and current-voltage characterization. Finally, Section 4 discusses the broader technical implications and highlights potential avenues for further investigation.

## 2. Experimental

The starting materials were <100> n-type silicon wafers with a resistivity of about 10–15 Ω cm. After the standard cleaning, the wafer was loaded into the multiple high-vacuum chamber system. The Al-laminated La_2_O_3_ film, ALO, was prepared as follows. The lanthanum oxide film of 3 nm thick was deposited on the wafer by sputtering. The sample was then transported to another chamber for Al deposition. After that, the sample was transferred back to the La_2_O_3_ deposition chamber for another 3 nm thick La_2_O_3_ film deposition. To compare the impact of Al-lamination, pure La_2_O_3_ of 5 nm thick was deposited in the same way. The sample was finalized with a thick Al layer for capping. Post-metal annealing (PMA) treatment was carried out in a nitrogen environment at a temperature of 300 °C for 25 min. Some of the samples underwent a rapid thermal annealing (RTA) in a nitrogen atmosphere at 400, 600, and 700 °C for 30 min, respectively.

To investigate the bonding structure of dielectric thin films, X-ray photoelectron spectroscopy (XPS) was performed using a Physical Electronics PHI 5600 system with a monochromatic Al Kα source (1486.6 eV). The carbon 1s peak at 284.6 eV served as a reference for calibration. Composition profiling was conducted through Ar^+^ ion sputtering, with an etch rate of approximately 0.26 nm/s. Fourier transform infrared (FTIR) spectra were recorded in absorbance mode at room temperature using a Bio-Rad FTS 6000 spectrophotometer, covering a wavenumber range of 500 to 4000 cm^−1^. Electrodes, ranging from 100 to 300 μm in diameter, were patterned via photolithographic techniques.

The electrical properties, including capacitance–voltage (C-V) and leakage current density-applied electric field (J-E) characteristics, were assessed using an Agilent B1500 semiconductor analyzer. All electrical measurements were conducted in a dark, electromagnetically shielded chamber.

## 3. Results and Discussion

**Figure 1:**
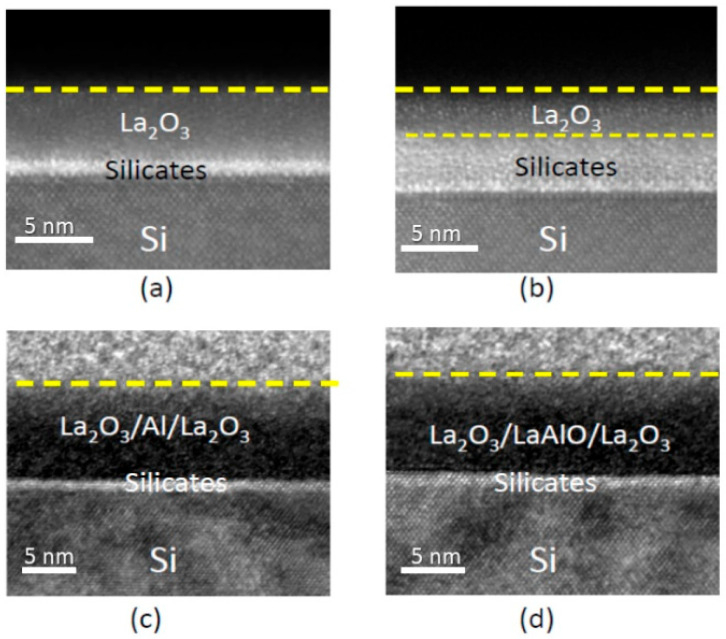
Cross-sectional TEM image of (**a**) as-deposited La_2_O_3_ oxide; (**b**) La_2_O_3_ oxide with 600 °C annealing; (**c**) La_2_O_3_ oxide with Al lamination; and (**d**) Al-laminated La_2_O_3_ with 700 °C thermal annealing.

Figure 1 depicts the cross-sectional TEM images of four different samples. As-deposited and thermally annealed La_2_O_3_ are shown for comparison. A thick interface silicate layer of over 2.5 nm was developed after thermal annealing at 600 °C. This layer induced various electronic instabilities and led to significant EOT degradation. In addition, high-resolution TEM further shows that the interfaces are rougher, which could lead to channel mobility degradation and local electric field fluctuation [23,27]. These interface degradations are attributed to oxidation and silicate formation at the interface [2,28]. The samples with Al-lamination exhibit a clear and well-defined interface with the silicon substrate. The interface sharpness is maintained even after thermal annealing at 700 °C, with the change in the interface thickness being almost negligible. This indicates that the ALO thin film and the silicon substrate have a low reactivity even at high temperatures. These phenomena will be examined in detail in the following XPS analysis. Their impact on the electrical characteristics will be discussed.

(a) **XPS Study**

**Figure 2:**
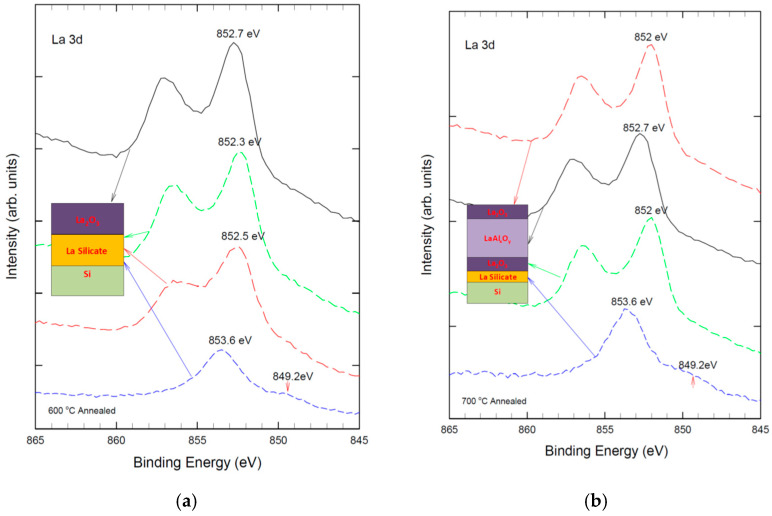
La 3d XPS spectra of: (**a**) La_2_O_3_ film with 600 °C thermal annealing; and (**b**) Al-laminated La_2_O_3_ film with 700 °C annealed at different depths.

Figure 2 depicts the La3d XPS spectra of pure La_2_O_3_ film and Al-laminated La_2_O_3_ film at different depths. As shown in Figure 2a, the pure La_2_O_3_ film exhibits a characteristic bimodal structure of the La 3d2/3 peak, which is attributed to the charge transfer between O (2p) and La 3d2/3 orbitals due to the unpaired d electrons of La. The main and satellite peaks are located at 851.4 eV and 855.2 eV, respectively [29,30]. The peaks are slightly shifted to a higher binding energy when sputtered closer to the La_2_O_3_/Si interface, which indicates the formation of silicate phases in the film [29,30]. The intensity of the La 3d peak decreases significantly, whereas the peak width increases due to the compositional variation in the silicate phases. This is because the La-O-Si bond has a higher binding energy than the La-O-La bond, as the electron density on O is more polarized towards Si. The Al-laminated La_2_O_3_ film shows a different behavior after thermal annealing (see Figure 2b). The La 3d2/3 peak splits into two peaks at 852.7 eV and 856.2 eV, respectively, which are higher than those of the pure La_2_O_3_ film. This is due to the formation of La-O-Al bonds, which have a higher binding energy than La-O-Si bonds, as Al is more electronegative than Si.

**Figure 3:**
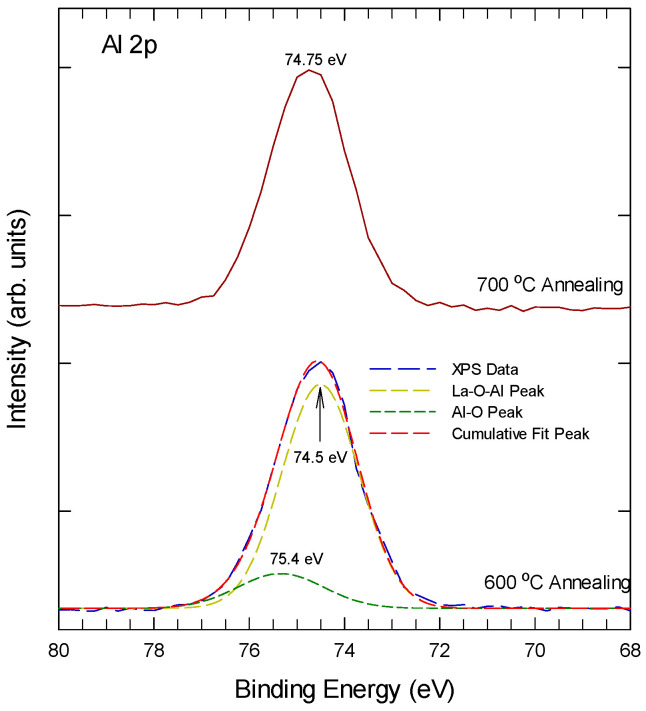
Al 2p spectra ALO with 600 °C and 700 °C rapid thermal annealing.

Figure 3 shows the Al 2p XPS spectra of Al-laminated La_2_O_3_ thin film at different annealing temperatures. For samples after 600 °C thermal annealing, the Al 2p can be decomposed into two sub-peaks at 75.4 ± 0.2 eV and 74.5 ± 0.2 eV. The Al 2p peaks at 75.4 ± 0.2 eV, a typical feature of the Al_2_O_3_ phase, which should arise from the reaction of elemental Al of the laminated layer with the as-sputtered O from the top La_2_O_3_ layer. The 74.5 eV should be due to the La-O-Al bonding, which is close to 74.9 eV of La-O-Al bonding of La_x_Al_2__−x_O_3_ deposited by ALD [31]. With 700 °C thermal annealing, the 75.4 eV is reduced to a minimum, indicating that most Al atoms now appear as La-O-Al bonding.

**Figure 4:**
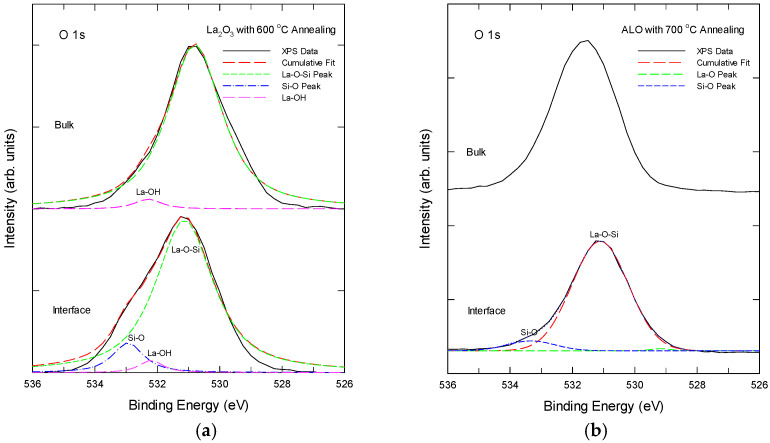
Oxygen 1s XPS of (**a**) the La_2_O_3_ sample with 600 °C annealing, and (**b**) the 700 °C annealed Al-laminated La_2_O_3_ film at different depths.

It is widely accepted that two different types of oxygen species can be distinguished from a solid. The lattice oxygen or metal-bonded oxygen is present in the form of O^2−^ at lower binding energy values and chemisorbed oxygen is present at higher binding energy values, which can be assigned to oxygen species with a lower electron density. The adsorbed oxygen species on the surface come from hydroxyl groups, water, and/or carbonate species [32,33]. As shown below, O 1s XPS could disclose further details of the bonding states of the La and Al in the samples and is worth detailed investigation. The O 1s XPS spectra of the La_2_O_3_ and ALO samples after thermal annealing are shown in Figure 4. Figure 4a shows the XPS spectra taken in the bulk region and at the La_2_O_3_/Si interface region for the La_2_O_3_ sample with 600 °C. The bulk region exhibits an O 1s peak at 530.8 eV, which is due to the La-O-Si bonding. A Gaussian fitting reveals a small component of a 532.4 ± 0.2 eV peak in the bulk, which is related to the La-O-H bonds or chemisorbed oxygen due to the hygroscopic nature of La_2_O_3_ [26,27]. At the interface, although the dominant peak was still around 530.8 eV, sub-peaks at around 532.4 ± 0.2 eV and 533 ± 0.2 eV were found with the peak fitting. The 533 ± 0.2 eV peak is due to the Si-O-Si bonds. Therefore, both the interface and the bulk have a significant silicate phase in the thermally annealed La_2_O_3_ film.

For ALO samples with thermal annealing, as shown in Figure 4b, the O 1s spectrum of both the bulk and interface displays a dominant peak at approximately 531.6 eV, which is attributed to the La-O-Al bonding, primarily due to the higher electronegativity of aluminum compared to lanthanum and silicon. No other peaks were found with Gaussian peak decomposition. At the ALO/Si interface, the O 1s spectrum can be decomposed into two sub-peaks with a dominant peak at 531.2 eV and a weak peak with an energy of 533.4 eV, respectively, which are attributed to the silicate and Si-O bonding [29,30,31]. As compared with the La_2_O_3_/Si interface in Figure 4a, the silicate and Si-O peaks are much weaker and were found in a very narrow region. No La-OH was found in the ALO samples.

**Figure 5:**
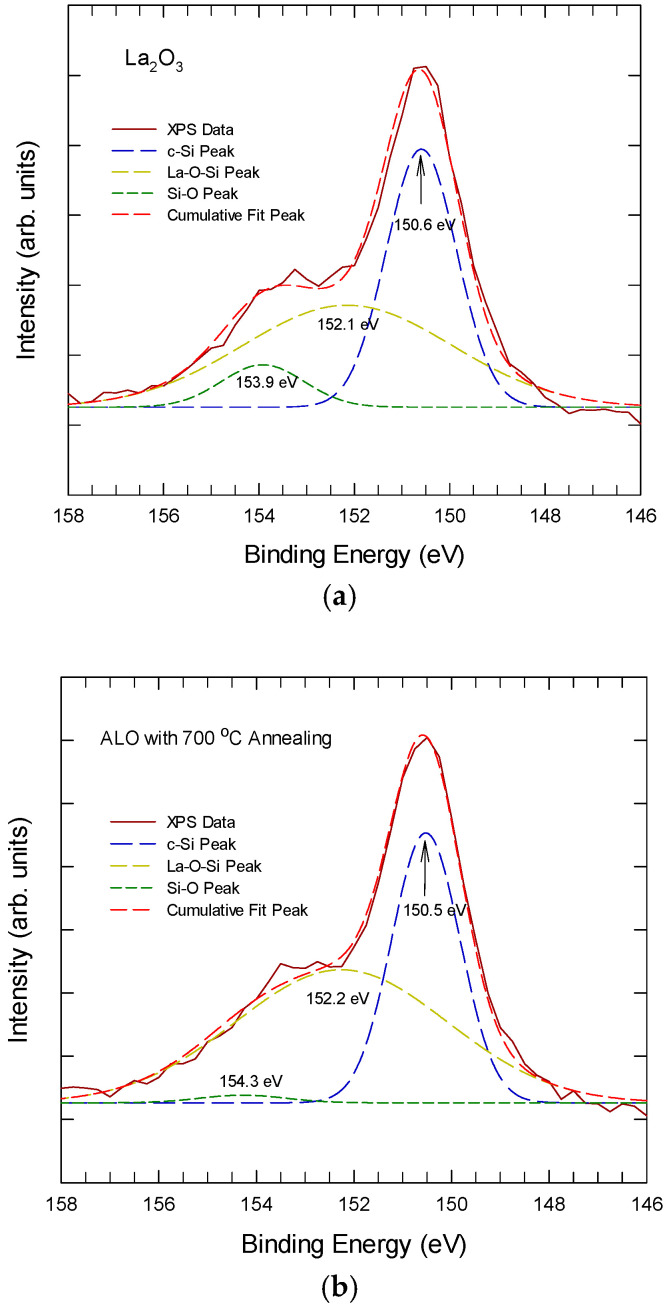
The Si 2s XPS spectra, taken from the interface region for (**a**) the La_2_O_3_ thin film and (**b**) the Al-laminated La_2_O_3_ thin film.

Figure 5 shows the Si 2s XPS spectra from the Si/La_2_O_3_ interface region for La_2_O_3_ and Al-laminated La_2_O_3_ thin films. The La_2_O_3_ sample exhibits a broad peak at the interface and further develops into the bulk of the lanthanum oxide. At the interface and after 600 °C thermal annealing, the broad Si 2s peak can be resolved into three sub-peaks at around 150.8 eV, 152 eV, and 154 eV. The sub-peak at 150.8 ± 0.3 eV is attributed to crystalline silicon, the sub-peak at about 152 ± 0.2 eV is attributed to silicate bonding, and the sub-peak at 154 ± 0.3 eV is attributed to Si-O bonding. For the ALO samples, the Si 2s peak at the interface is much weaker and confined to a narrow region near the interface. The 152 ± 0.2 eV sub-peak increases slightly after annealing at 700 °C, while the Si-O peak remains undetected in the ALO even after 700 °C annealing. This suggests that both the as-deposited Al layer and the LaAl*_x_*O*_y_* layer can suppress the outward diffusion of Si from the substrate, leading to a reduced amount of Si-related bonding. These findings again agree with the TEM pictures shown in Figure 1.

According to the XPS results, the pure La_2_O_3_ sample with annealing has a higher number of OH groups or water molecules, which reflects the hygroscopic nature of La_2_O_3_ [34]. The OH group and water molecules can cause interface oxidation, facilitating the incorporation of substrate Si in the bulk La_2_O_3_ [2]. This finding is consistent with the TEM results, which show that La_2_O_3_ with thermal annealing leads to significant growth in the low-k interface silicate layer. For the Al-laminated La_2_O_3_ composite film (ALO) proposed in this work, the thermal annealing has less effect on the interface layer growth and the formation of the bulk silicate phase. This could be attributed to the multiple roles of the Al-laminated layer. First, the layer can effectively block the diffusion of oxygen or the OH group into the interface. Second, oxygen should participate in the lanthanum aluminate formation under certain annealing conditions. Third, the oxidation of the laminated Al layer could give rise to the interface silicon oxide scavenging. Because of these actions, the proposed method should be better than the direct deposition of LaAlO_3_ on the silicon substrate [35]. Although LaAlO_3_ was found to be thermally stable and have an extremely low leakage current, the SiOx phase or interface layer growth was still observed at the LaAlO_3_/Si interface with vacuum thermal annealing at 500 °C or higher [35].

(b) **Fourier Transform Infrared (FTIR) spectra**

**Figure 6:**
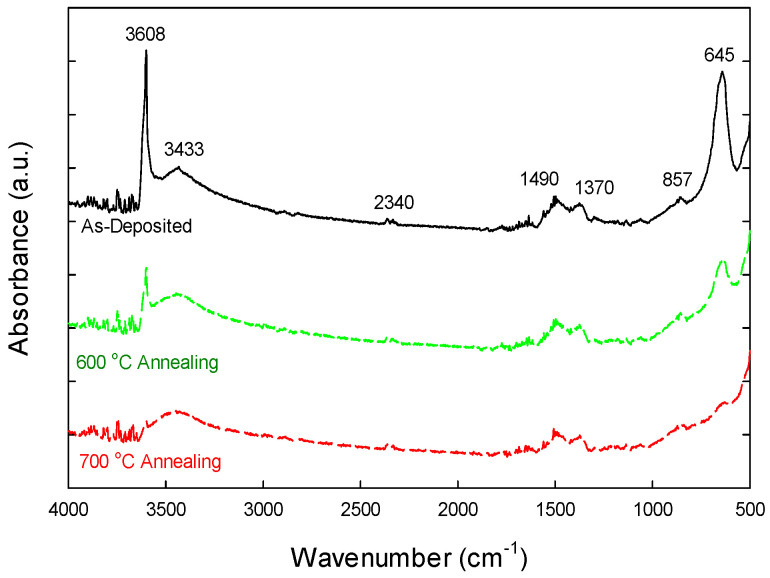
Fourier Transform Infrared (FTIR) spectra of as-deposited La_2_O_3_-Al_2_O_3_ laminate and those subjected to annealing at 600 °C and 700 °C.

To confirm the involvement of hydroxyl groups in the thermally annealing effect on the lanthanum aluminate formation, Fourier Transform Infrared (FTIR) spectra were taken on various samples, spanning a wavenumber range of 4000–500 cm^−1^. Figure 6 illustrates the typical FTIR spectra of the as-deposited Al-laminated La_2_O_3_ and those subjected to annealing at 600 °C and 700 °C. The FTIR spectra of La_2_O_3_ typically exhibit lanthanum-oxygen (La-O) bending vibrations in the 530–645 cm^−1^ range. The as-deposited sample exhibits a distinct absorption band at 3608 cm^−1^, attributed to the stretching vibrations of the OH group associated with La^3+^ cations, while a pronounced peak near 645 cm^−1^ corresponds to the bending vibration [36,37,38]. Broadband peaking at 3433 cm^1^ indicates the stretching vibrations of O–H bonds, likely due to moisture adsorption on the sample surface [38,39]. The peaks in the range of 1490–1370 cm^−1^, along with a feature at 857 cm^−1^, could originate from the asymmetric and symmetric stretching of the COO^−^ functional groups and C–O bending vibrations, respectively, which stem from atmospheric surface carbonate species [38]. Other than the O-C-O bending in carbonate species, the 857 cm^−1^ peak may also be associated with Al-O or La-O bonding. This requires further experimental confirmation, however. The absorption feature at 2340 cm^−1^ is attributed to the asymmetric stretching vibrations of carbon dioxide (CO_2_) molecules adsorbed onto the sample or present within the optical path of the FTIR instrument. Notably, no SiO_2_ peaks—typically found around 1060 cm^−1^—were detected [39], indicating a significant reduction in the formation of Si-O-La bonds within La_2_O_3_.

Upon thermal annealing, the 645 cm^−1^ peak diminishes in samples annealed at 600 °C and 700 °C, suggesting the formation of La-O-Al bonding. The peaks at 3608 cm^−1^ are markedly reduced due to the removal of OH groups, with those observed at 700 °C being nearly completely eliminated. These findings confirm that the laminated aluminum layer effectively mitigates the hygroscopic nature of lanthanum oxide and inhibits silicate layer formation during high-temperature annealing.

(c) **Electrical Characterization**

**Figure 7:**
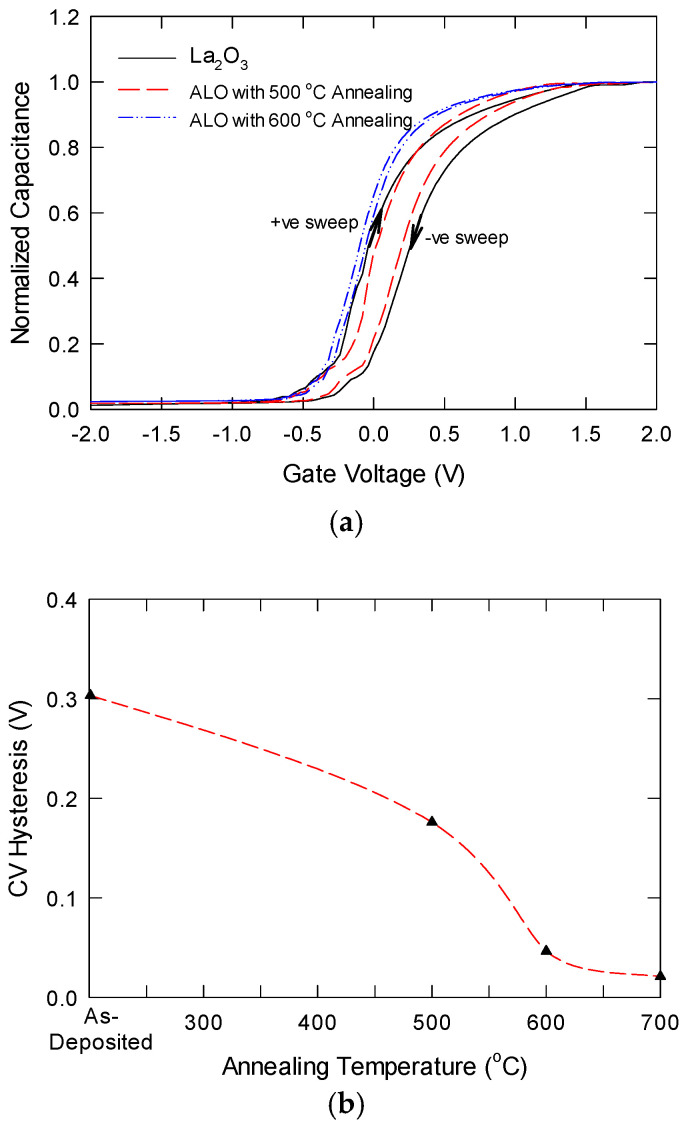
(**a**) Comparison of bidirectional high-frequency (1 MHz) capacitance-voltage characteristics for La_2_O_3_, ALO with 600 °C, and ALO with 600 °C annealing; (**b**) plot of CV hysteresis as a function of annealing temperature.

Figure 7a shows the bidirectional C-V characteristics of La_2_O_3_, and ALO annealed at different temperatures. Since the samples have different thicknesses and different k values, the samples would have different capacitance values. To ensure a fair comparison, we normalized the C-V with the oxide capacitance (C_ox_) value. The equivalent dielectric constant estimated from the C_ox_ values was about 17.5, 19.2, and 23.1, respectively, for the La_2_O_3_ sample, ALO with 600 °C annealing, and ALO with 700 °C annealing. The k-value of the ALO with 700 °C is close to the reported value for LaAlO_3_ thin films, and this suggests that the ALO/Si interface growth can be almost neglected in this situation. This result is better than directly depositing stoichiometric LAO on silicon with a similar thermal budget [29]. For La2O3 and ALO samples with 600 °C annealing, hysteresis was observed for the bidirectional sweeps. As shown in Figure 7b, La_2_O_3_ was found to have a larger hysteresis, which was usually attributed to oxygen vacancies. In contrast, the hysteresis in ALO with 600 °C annealing was smaller. Other than the oxygen vacancies, here, the hysteresis in the ALO sample should be due to the incomplete reaction of Al when converting into the LaAl*_x_*O*_y_* structure. The metallic Al can trap electrons during a positive sweep, resulting in a negative flatband shift. The hysteresis for ALO with 700 °C annealing is negligible (the two curves are almost overlapped), and the more negative flatband should be due to the removal of position charges in the LaAl*_x_*O*_y_* film. This implies that the as-grown LaAl*_x_*O*_y_* layer has a very low oxide trap density. Meanwhile, the transition slope between accumulation and strong inversion reflects the interface trap density. As shown in Figure 7a, the sample annealed at 700 °C has the sharpest transition, implying it has the lowest interface trap density. Furthermore, a small hump is observed in the C-V curves of the La_2_O_3_ sample and ALO with 600 °C annealing, which could be due to the presence of interface traps.

**Figure 8:**
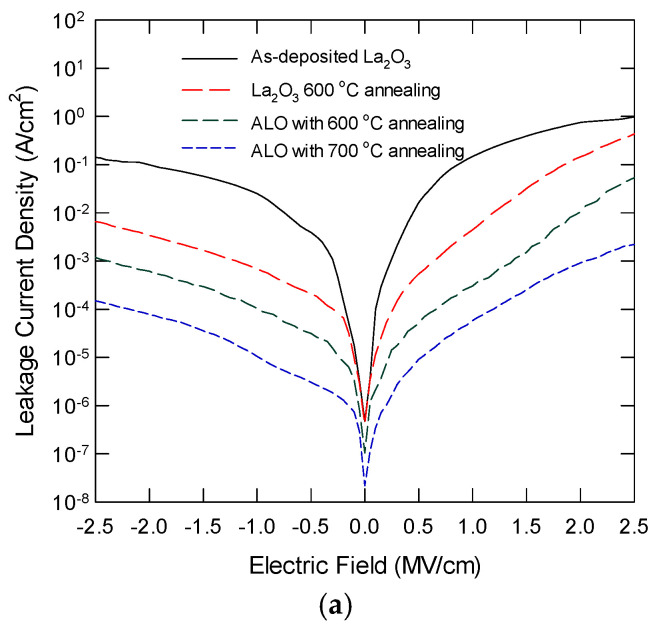

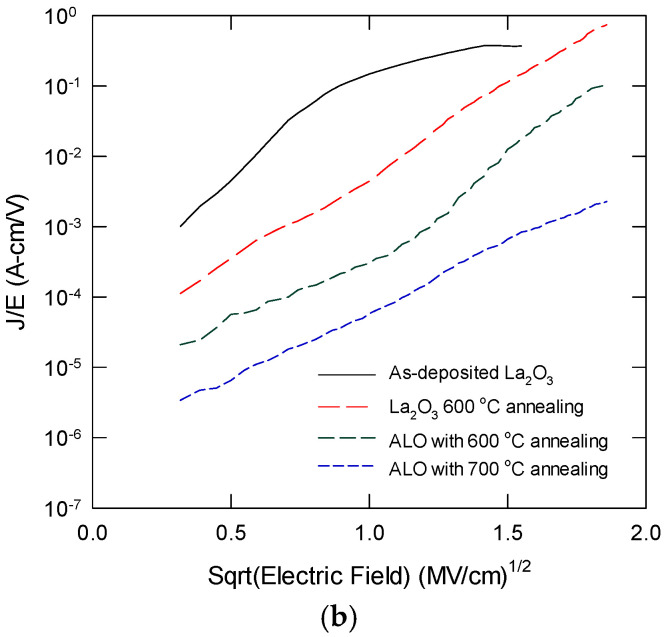
(**a**) Leakage current characteristics of La_2_O_3_ with 600 °C, and ALO with 600 °C and 700 °C annealing; (**b**) Poole–Frenkel plot of the forward leakage characteristics for various samples.

Figure 8a plots the leakage current density as a function of the applied electric field for ALO with thermal annealing at different temperatures. The leakage characteristics of pure La_2_O_3_ are also shown for comparison. La_2_O_3_ always has the largest leakage current under the same electric field, and it also has a larger slope. These results were due to the significant trap-assisted current conduction via the oxide defects, such as oxygen vacancies [27]. For ALO samples with proper thermal annealing, the leakage current is reduced by one to two orders of magnitude. For ALO annealed at 600 °C, a semi-saturation plateau is observed, which can be attributed to charge localization at the non-oxygen bridging Al atoms. ALO with 700 °C annealing has the smallest leakage current, and the J-E slope is also smaller, indicating a significant reduction in trap-assisted conduction. Figure 8b shows the Poole–Frenkel plot of the leakage current characteristics in the forward region. The 700 °C annealed ALO follows the PF relationship in the applied forward bias. La_2_O_3_ film ALO with 600 °C shows a rapid increase in the leakage current for *E*^1/2^ at around 1.1 and 1.3 (MV/cm)^1/2^, respectively. This implies the involvement of trap-assisted current conduction. This performance improvement is attributed to the incorporation of Al atoms to form a high-quality LaAl*_x_*O*_y_* layer that contains fewer defects. Another factor leading to the low leakage current could be the large energy band offset values at the La_2_O_3_/Si interface. Without a notable silicate layer and with a high amount of interface trap density, the conduction band offset should be close to that of the native La_2_O_3_/Si.

**Figure 9:**
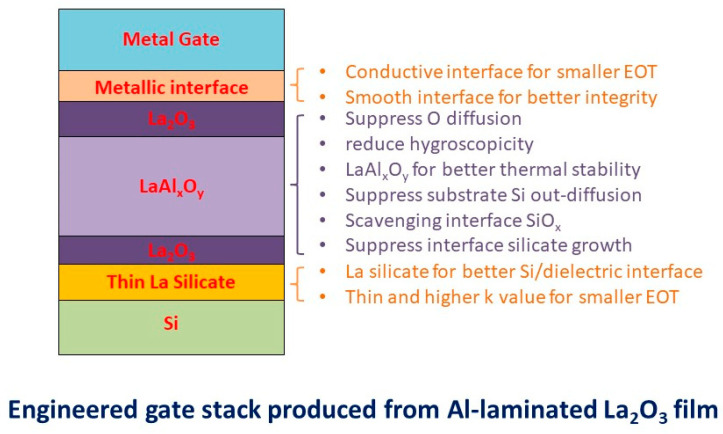
Features achieved with the La_2_O_3_/LaAl*_x_*O*_y_*/La_2_O_3_ prepared through the thermal annealing of an Al-laminated La_2_O_3_ stack.

The structure and the possible benefits of the proposed Al-laminated La_2_O_3_ stack engineering can be inferred from the XPS and electrical characteristics discussed above. Figure 9 illustrates the possible resultant film structure of the Al/La_2_O_3_/Al/La_2_O_3_/Si stack after high-temperature annealing. The stack has several improved material and electrical characteristics compared to its simple La_2_O_3_ or LaAlO_3_ counterparts. The laminated Al layer has two functions during thermal annealing. First, it blocks the diffusion of O or OH groups into the La_2_O_3_/Si interface. Second, it forms LaAl*_x_*O*_y_* by consuming O and OH residuals and the remote scavenging of interface silicon oxide, which were incorporated in the La_2_O_3_ or formed during deposition. The LaAl*_x_*O*_y_* formation process and the remote scavenging mechanisms are presented as follows.

The laminated Al can be readily oxidized with the residual O and OH groups to form Al-O, Al-OH bonding as follows:

2Al + 3[O] → Al_2_O_3_ (1)

Al + [OH] → Al-OH(2)

Since the bottom La_2_O_3_ layer is so thin, a remote scavenging reaction is also possible from the entropy perspective. The reaction is as follows:

3SiO_2(interface)_ + 4Al_(laminated layer)_ → 3Si_(substrate)_ + 2Al_2_O_3(laminated layer)_ (3)

The aluminum oxide generated in the above reactions can further react with the lanthanum oxide via calcination, as follows:

La_2_O_3_ + Al_2_O_3_ → 2LaAlO_3_ (4)

In addition to the remote scavenging process during the high-temperature annealing presented in (3), the rest of the above chemical reactions should also prevent Si atoms from diffusing from the substrate into the La_2_O_3_ film and reduce the interface oxidation. It was suggested that the interface growth was mainly due to the existence of a SiO_x_ precursor [2]. In the current proposed process, the calcination reaction presented in (5) can be significantly suppressed because only a few interfacial silicon oxide groups are present. This, in turn, limits the interface layer’s growth. The formation of silicate layers can lead to a decrease in the k value of the material and cause phase separation effects when the film is exposed to high temperatures.

La_2_O_3_ + *x*SiO_2_ → 2LaSi*_x_*O*_y_* +(3 + 2*x* − 2*y*)O (5)

When compared to the direct deposition of LaAlO_3_ on the Si, a thin buffer layer of SiO_2_ or a La silicate layer can provide better dielectric/Si interfacing to reduce the interface trap density. A similar technique was adopted in the Hf-based dielectric for CMOS circuit manufacturing. Hence, the present La_2_O_3_/LaAl*_x_*O*_y_*/La_2_O_3_ structure has the advantage of a lower interface trap density while maintaining a smaller EOT. The LaAl*_x_*O*_y_* middle layer, formed after thermal annealing at 700 °C, is more thermally stable and contains fewer defects. It also serves as a blocking layer for O in-diffusion and Si out-diffusion. However, the La_2_O_3_/LaAl*_x_*O*_y_* stack results in a significant reduction in the gate leakage current for the larger band offsets between La_2_O_3_ and Si and the low current leakage of the LaAl*_x_*O*_y_* layer. The top La_2_O_3_ layer should have a better interface with TaN or W than the metal gate/LaAlO_3_ [35]. This issue is worth further in-depth investigation.

## 4. Conclusions

The aim of this research is to address the challenge of reducing the equivalent oxide thickness (EOT) of gate dielectrics for advanced devices. The quality and stability of the dielectric/Si interface are crucial to achieving this goal. In this context, a new method for fabricating lanthanum-based dielectric films with excellent properties was proposed. By inserting an Al-laminated layer into La_2_O_3_ thin films and following this with a high-temperature rapid thermal annealing, a La_2_O_3_/LaAl_x_O_y_/La_2_O_3_ sandwich dielectric structure was obtained. The Al layer and the LaAl_x_O_y_ layer act as barriers for oxygen and hydroxy migration, as well as Si out-diffusion, during the annealing process, as confirmed by TEM, XPS, and FTIR measurements. This prevents the formation of a thick silicate layer at the La_2_O_3_/Si interface and enhances the integrity of the dielectric films. The electrical characterization showed that the stack films had much lower interface and lower oxide trap densities than the pure La_2_O_3_ sample. This novel method provides a simple and effective way to engineer high-quality lanthanum-based dielectrics with the ability to control the dielectric/substrate interaction. The proposed method should be compatible with the standard CMOS process. The only issue is that the annealing thermal budget may be a slightly too high. This can be overcome with rapid thermal annealing and the gate-first process [2].

## Data Availability

The original contributions presented in the study are included in the article, further inquiries can be directed to the corresponding author.
